# Magnesium Oxide Nanoparticles: A New Frontier in Antiviral Therapy Against Herpes Simplex Virus Type 1

**DOI:** 10.1155/av/3088529

**Published:** 2025-08-17

**Authors:** Abdulhussain Kadhim Jwaziri, Zahra Salavatiha, Seyed Jalal Kiani, Pegah Khales, Masoud Vazirzadeh, Ahmad Tavakoli

**Affiliations:** ^1^Department of Microbiology, College of Medicine, University of Kerbala, Karbala, Iraq; ^2^Department of Virology, School of Medicine, Iran University of Medical Sciences, Tehran, Iran; ^3^Department of Bacteriology and Virology, School of Medicine, Shiraz University of Medical Sciences, Shiraz, Iran; ^4^South Texas College, McAllen, Texas, USA; ^5^Research Center of Pediatric Infectious Diseases, Institute of Immunology and Infectious Diseases, Iran University of Medical Sciences, Tehran, Iran

**Keywords:** herpes simplex virus, HSV, magnesium oxide, MgO, nanoparticles

## Abstract

**Background and Aims:** Herpes simplex virus Type 1 (HSV-1) causes a wide spectrum of diseases in humans, including skin and mucosal ulcers, encephalitis, and keratitis. Acyclovir is regarded as the gold standard for treating infections with this virus. However, there are certain drawbacks to using this drug, such as its ineffectiveness against treatment-resistant virus strains. Therefore, the development of novel and effective drugs to combat this virus is urgently needed. The present work aims to explore the efficacy of magnesium oxide nanoparticles (MgONPs) against HSV-1 in vitro as a potential novel antiviral agent.

**Methods:** MgONPs were characterized by X-ray diffraction, energy-dispersive X-ray spectroscopy, field-emission scanning electron microscope, ultraviolet-visible spectrophotometry, Fourier-transform infrared spectroscopy, dynamic light scattering, and zeta potential. To assess the cytotoxic effects of MgONPs on Vero cells, the neutral red uptake assay was used. The effects of MgONPs at nontoxic concentrations on HSV-1 were then examined using a quantitative real-time PCR assay.

**Results:** No toxic effect was observed in all used concentrations of MgONPs (up to a concentration of 1000 μg/mL). Three-hour incubation of HSV-1 with MgONPs at concentrations of 900 and 1000 μg/mL resulted in a remarkable decrease in viral load with an inhibition rate of 93.6% and 96.8%, respectively. The results from the posttreatment assay also showed that MgONPs at concentrations of 300 and 1000 μg/mL led to a significant decrease in viral load with an inhibition rate of 99.5% and 99.7%, respectively.

**Conclusion:** MgONPs can exert their inhibitory effects on HSV-1 in a dose-dependent manner, both directly and through interfering with the replication cycle of the virus.

## 1. Introduction

Herpes simplex virus Type 1 (HSV-1) belongs to the subfamily Alphaherpesvirinae of the Herpesviridae family with a relatively high prevalence of infection and is particularly prone to causing numerous disorders in humans (1). HSV-1 infection is primarily acquired orally in children and frequently appears as cold sores, but it can also cause more significant neurological, ocular, and mucocutaneous problems. Adults who were not infected orally as children can get genital HSV-1 infection. Sexual transmission of HSV-1 infection has become more common, especially in high-income nations, and it is currently the most common cause of first-episode genital herpes in a number of these nations. Almost 4 billion people, or two-thirds of the world's population between the ages of 0 and 49, were infected with HSV-1 in 2020, primarily orally, with over 120 million new cases reported in this year [1, 2].

Acyclovir, valacyclovir, and famciclovir are nucleoside analogs that target the viral DNA polymerase and are among the first-line treatments for HSV infections [3]. The gold standard for treating HSV-1 infections is still acyclovir, a guanosine analog with minimal toxicity and good selectivity [4]. Systemic treatment for HSV infections, such as labial and genital herpes, involves the use of acyclovir. For the treatment of HSV, other nucleoside analogs such as famciclovir and trifluridine are utilized in addition to acyclovir and its prodrug valacyclovir [5]. However, these medications have significant drawbacks, including resistance, insufficient suppression, low bioavailability, and short half-life [6, 7]. Currently, no drug is available to remove a latent infection, and the extended therapeutic use of antivirals in immunocompromised individuals can contribute to the occurrence of treatment failure due to the emergence of antiviral-resistant virus strains [8].

Nanotherapeutics could revolutionize the development of antiviral medications and solve problems with strain-specific targeting, resistance, new viruses, and incurable viral diseases [9]. It makes use of nanoparticles (NPs) between 1 and 100 nm in size as a way for drug delivery, infectious disease diagnostics, and therapy [9–11]. There are two types of therapeutic NPs: inorganic (such as metal NPs) and organic (such as polymeric, liposomes, micelles, and ferritin). For a range of medical disorders, both kinds of NPs have shown efficacy in preclinical research and clinical settings [12, 13]. Since metals can “attack” multiple targets on viruses with little effect on the later development of resistance, the use of metal NPs as antiviral medicines has expanded quickly in recent years [9, 14].

NPs have emerged as promising alternatives due to their unique physicochemical properties and ability to target bacteria through multiple mechanisms, reducing the likelihood of resistance development [15]. Nanomaterials, particularly metallic NPs functionalized with sulfonates or polyphenols such as tannic acid, offer promising alternatives due to their ability to inhibit viral entry and replication through diverse mechanisms, such as physical blocking or disruption of the viral capsid [16]. NPs, such as silver, gold, zinc oxide, and carbon-based nanomaterials, exhibit unique physicochemical properties that enable them to inhibit HSV-1 at various stages of its life cycle, including viral attachment, entry, replication, and cell-to-cell spread. Furthermore, NPs serve as effective nanocarriers for ACV, enhancing its bioavailability, reducing side effects, and enabling targeted delivery [6].

Magnesium oxide NPs, or MgONPs, stand out among other metal oxide NPs because they are less harmful to the host and have a number of advantageous characteristics. MgONPs have several qualities, including low toxicity, biodegradability, biocompatibility, strong antimicrobial activity, biomedical applications, high chemical stability, electrical permeability, and photocatalytic activity. They are also inexpensive and easily accessible. Furthermore, MgO is currently regarded by the United States' Food and Drug Administration (FDA) as a substance that is safe for human ingestion [17].

Some research has been performed on MgONPs' antifungal and antibacterial qualities, and data are accessible in this area. Nevertheless, only one study so far examined the antiviral effects of MgONPs and evaluated their antiviral efficacy against foot and mouth disease virus (FMDV), which is another viral infection that affects cattle. As a result, there is insufficient evidence known about MgONPs' antiviral effects, and more research is needed in this field. The purpose of this work is to examine the in vitro activity of MgONPs against HSV-1 as a possible novel antiviral drug, given the significance and high prevalence of HSV-1 and the related health and economic issues they present worldwide.

## 2. Materials and Methods

### 2.1. Characterization of MgONPs

US Research Nanomaterials Inc. provided highly purified MgO nanopowders (> 99%). The X-ray diffraction (XRD) (PHILIPS PW1730) with Cu-Kα radiation (*λkα* = 1.54 Å) was used to examine the crystal structure, chemical composition, and purity of the MgONPs. Using Fourier-transform infrared spectroscopy (FTIR), the surface functional group in MgONPs was assessed in the 450–4000 cm^−1^ range. Potassium bromide (KBr) and MgONP powders were mixed in a ratio of 1:19. The samples underwent analysis using FTIR (360 Nicolet AVATAR spectrometer, Thermo Scientific, USA). To assess the size and shape of the NPs, a field-emission scanning electron microscope (FESEM) (MIRA3TESCAN-XMU) was used. Moreover, energy-dispersive X-ray (EDX) spectroscopy was used to assess the sample's chemical purity. Moreover, the surface area of the MgONPs was examined using Brunauer–Emmett–Teller (BET) (BELsorp-MINI II, BEL, Japan) after degassing at 175°C for 90 min in streaming nitrogen, followed by measuring the adsorption isotherm of nitrogen gas at a temperature of 77 K. The optical absorption range of MgONPs was measured using an ultraviolet-visible (UV-vis) spectrophotometer (AVaSpec 2048 TEC) in the wavelength range of 250–800 nm. The dynamic light scattering (DLS) method was used to evaluate the MgONPs' hydrodynamic measure and zeta potential at room temperature.

### 2.2. Cells and Viruses

African green monkey kidney (Vero) cells were grown in high-glucose Dulbecco's Modified Eagle's Medium (DMEM) (Gibco, Invitrogen, USA) supplemented with 10% heat-inactivated fetal bovine serum (FBS) (Gibco, Invitrogen, USA), 2 mM l-glutamine (Merck, Germany), 100 U/mL penicillin, and 100 μg/mL streptomycin (Sigma-Aldrich, USA) at 37°C with 5% CO_2_. The HSV-1 KOS strain was used to measure antiviral activity. After the virus was propagated using Vero cells, the Reed and Muench method was used to determine the propagated viral stock infectious titer as TCID_50_ mL^−1^. Following titration, the viral stock was aliquoted in sterile microtubes and kept at −70°C for further use.

### 2.3. Determination of Cell Cytotoxicity

The cytotoxicity of MgONPs was evaluated through the neutral red uptake assay, a method used to measure cell viability by quantifying the cytotoxic effects of MgONPs in vitro. This assay depends on the capacity of viable cells to incorporate and bind neutral red, a weakly cationic dye, within their lysosomes [18]. Consequently, cytotoxicity is indicated by a concentration-dependent decrease in neutral red uptake following exposure to MgONPs. A 96-well microtiter plate was seeded with 1.5 × 10^4^ Vero cells per well. The growth media were removed from the wells, and different concentrations of MgONPs (100–1000 μg/mL) were added to them after 24 h of incubation at 37°C in a humid environment with 5% CO_2_. The plate was incubated for 48 h at 37°C in a humidified incubator with 5% CO_2_. Following the incubation period, the growth medium containing various MgONP concentrations was removed. After adding 100 μg/mL of neutral red to each well, the cells were incubated for 3 h. The integrated dye was released from the cells following the addition of desorption solution (1% glacial acetic acid solution, 50% EtOH, and 49% H_2_O) to each well. A microplate reader (Hiperion MPR 4+, Roedermark, Germany) was used to measure the absorbance at a test wavelength of 550 nm. Data are presented as a percentage of cell viability relative to untreated cells (negative control, set at 100% viability).

### 2.4. Determination of Antiviral Activity

#### 2.4.1. Virucidal Assay

In brief, 100 μL of HSV-1 suspension (100 TCID_50_/mL) was incubated with 100 μL of MgONPs in two nontoxic concentrations for 3 h at 37°C in a humidified 5% CO_2_ atmosphere. In parallel, the same amount of the viral solution was incubated with cell culture media without MgONPs and was used as a virus control in this assay. A 96-well microtiter plate was seeded with 1.5 × 10^4^ Vero cells per well. After 24 h of incubation at 37°C in 5% CO_2_, Vero cell monolayers were then treated with the above mixtures and incubated for an additional hour at 37°C. Following incubation, the supernatant was removed, and the cells were gently washed three times with PBS to eliminate any nonabsorbed viruses. Fresh DMEM media containing 2% FBS was then added, and the cells were incubated at 37°C for 48 h, and the viral load of HSV-1 was calculated using the quantitative real-time PCR (qPCR) assay.

#### 2.4.2. Cell Posttreatment Assay

Monolayers of Vero cells were prepared in a 96-well plate and infected with 100 TCID_50_/mL of HSV-1 solution for one hour at 37°C. Following the infection, the cells were rinsed with PBS to remove any noninternalized viruses. Different nontoxic concentrations of MgONPs were then added to the infected Vero cells, and the plate was further incubated for 48 h at 37°C with 5% CO_2_. Cell and virus controls were included in the experiment using the same conditions, and the viral load of HSV-1 were calculated using the qPCR assay.

### 2.5. qPCR Analysis

qPCR was used to confirm the impact of MgONPs on HSV-1 infection of Vero cells. HSV viral DNA was extracted from the supernatants of virus-infected Vero cells taken at virucidal and cell posttreatment experiments using the BehPerp Viral Nucleic Acid Extraction Kit (BehGene Biotechnology, Iran) according to the manufacturer's guidelines. The forward, reverse, and probe sequences targeting the UL30 gene of HSV-1 were 5′-ATCGGCGAGTACTGCATACA-3′,5′-GAGCTCCAGATGGGGCAA-3′, and 5′-HEX-ATTCCCTGCTGGTGGGCCA-BHQ1-3′, respectively, with an amplicon size of 75 bp. The real-time PCR was performed in a final volume of 25 μL reaction including 12.5 μL of the RealQ Plus 2x Master Mix for Probe, without ROX™ (Ampliqon, Denmark), 2 μL of forward primer (10 μM), 1 μL of reverse primer (10 μM), 1 μL of probe (10 μM), 5 μL of template DNA, and 3.5 μL of ddH_2_O. The experiment was carried out using the Rotor-Gene Q instrument (QIAGEN, Germany) under the following conditions: 10 min for initial denaturation at 95°C, followed by 40 cycles of 10 s at 95°C and 30 s at 60°C [19].

A reference standard was prepared by using the pUC57 vector containing the corresponding specific viral sequence. Tenfold dilutions equivalent to 10^−1^–10^−10^ concentrations of the synthesized plasmid were prepared to generate a calibration curve and to be run in parallel with the test samples. The copy number of the plasmid was calculated using an online DNA copy number calculator (Technology Networks). The limit of detection (LOD) of this real-time PCR was 3 copies/μL. Each run had a positive and a negative control, and all reactions were carried out in triplicate.

### 2.6. Statistical Analysis

All statistical analyses were performed using the statistical software SPSS, Version 22.0 (IBM SPSS Statistics, Chicago, IL, USA). The statistical significance of the data was assessed by conducting a one-way analysis of variance (ANOVA), followed by a Dunnett's post hoc test to compare multiple groups against a control group. All *p* values ≤ 0.05 were considered statistically significant. All graphs and charts were created using GraphPad Prism 8.

## 3. Results

### 3.1. Characterization of MgONPs

**Figure 1:**
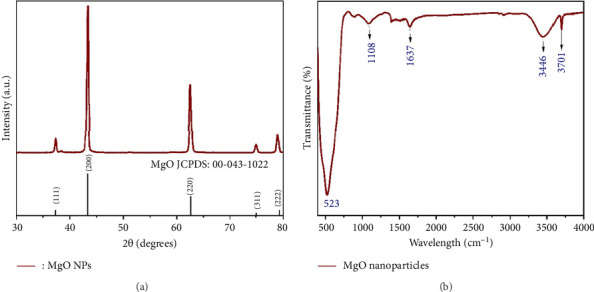
(a) X-ray diffraction (XRD) pattern of magnesium oxide nanoparticles (MgONPs), showing characteristic peaks at 36.9°, 42.9°, 62.2°, 74.6°, and 78.6°, confirming the cubic structure of MgO, with the highest intensity peaks at 42.9° and 62.2° (Miller indices 200 and 220, respectively). (b) Fourier-transform infrared (FTIR) spectra of MgONPs, displaying absorption bands at 3446 and 1637 cm^−1^ (O-H stretching), 1108 cm^−1^ (O-H bending), 3701 cm^−1^ (hydroxyl groups), and 520 cm^−1^ (MgO vibrations) in the wavelength range of 480–4000 cm^−1^.

Figure 1(a) presents the XRD pattern of the synthesized MgONPs. Comparison of the XRD pattern of MgONPs with the standard pattern reveals characteristic peaks of MgO at 36.9°, 42.9°, 62.2°, 74.6°, and 78.6°, confirming successful synthesis. According to JCPDS no. 43–1022, the highest intensity peaks occur at 2*θ* values of 42.9° and 62.2°, with Miller indexes of 200 and 220, respectively, indicating the cubic structure of the single-phase MgO. Utilizing the Debye–Scherrer formula, the average crystallite size of the produced MgONPs was estimated, yielding a calculated particle size ranging from 40 to 65 nm. These findings align with previous studies on MgONP synthesis [20].

Figure 1(b) depicts the FTIR spectra of MgONPs. This analysis aimed to identify the different functional groups of MgONPs by detecting characteristic peaks within the wavelength range of 480–4000 cm^−1^. The bands observed at 3446 and 1637 cm^−1^ are associated with moisture content (O-H stretching bond vibration) present in either the analyzed sample or the precursor solution. As reported in various studies, it is widely recognized that NPs such as MgO possess a high specific surface area due to their elevated surface-to-volume ratio. Consequently, when exposed to the atmosphere, these NPs can readily absorb H_2_O molecules [21–23]. In addition, the bond vibrations observed at approximately 1108 cm^−1^ correspond to O-H bending vibrations. The absorption peak at 3701 cm^−1^ further indicates the presence of hydroxyl groups on the surface of MgO particles, representing a single OH species [24]. Moreover, the broad peak observed at lower frequencies near 520 cm^−1^ is characteristic of MgO vibrations, which is consistent with the findings reported in other studies [25–27].

**Figure 2:**
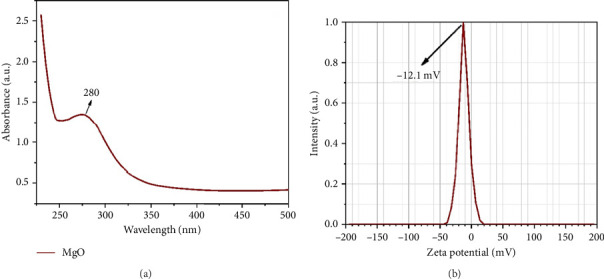
(a) Ultraviolet-visible (UV-vis) absorption spectrum of MgONPs, showing a distinct absorption peak at 280 nm within the 200–500 nm wavelength range, confirming the optical properties of MgONPs. (b) Zeta potential distribution of MgONPs, ranging from −40 to +20 mV with a peak at −12.1 mV, indicating good stability and negative surface charge of the nanoparticles in aqueous dispersion.

**Figure 3:**
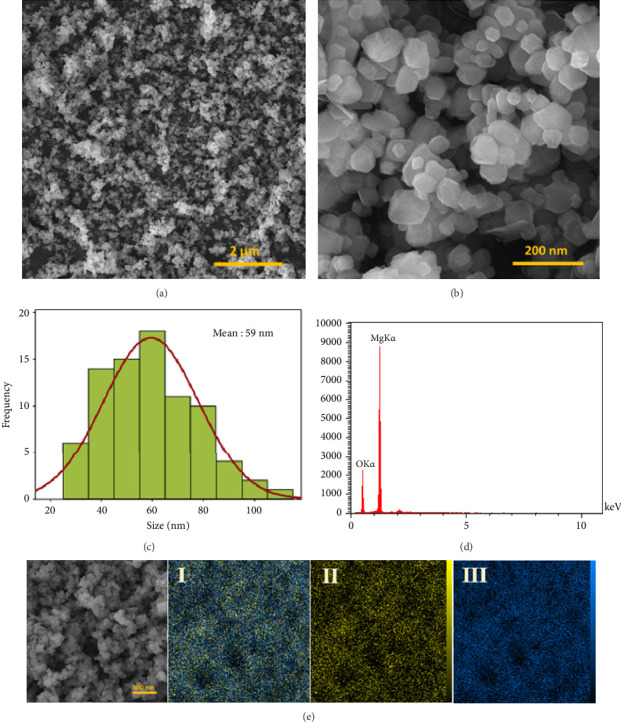
(a, b) Field-emission scanning electron microscopy (FESEM) images of MgONPs at 10 and 100 kx magnifications, revealing polyhedral crystalline nanoparticles with some agglomeration and well-defined hexagonal facets. (c) Size distribution histogram of MgONPs, showing an average diameter of approximately 59 nm, consistent with XRD results. (d) Energy-dispersive X-ray spectroscopy (EDX) spectrum, confirming the presence of magnesium (Mg) and oxygen (O) without impurities. (e) Elemental mapping images, illustrating the distribution of magnesium (blue) and oxygen (yellow) atoms, verifying the composition of MgONPs.

The optical characteristics of MgONPs were assessed utilizing a UV-vis spectrophotometer.  Figure 2(a) presents the UV-vis absorption spectroscopy of MgONPs, depicting absorbance as a function of wavelength within the range of 200–500 nm. The presence of MgONPs was verified by the distinct absorption peak observed at 280 nm. The obtained UV-vis absorption spectra align with the expected absorption band range of MgONPs, falling within the nonvisible light range, which is consistent with previous literature findings [26, 28, 29]. The zeta potential range for the MgONP sample was −40 to +20 mV, with the peak observed at −12.1 mV, indicating good stability of the dispersed MgONPs in water (Figure 2(b)). However, the negative zeta potentials indicate that the surfaces of MgONPs carry a negative charge [29]. The surface topography, morphology, and size distribution of MgONPs were examined using the SEM technique at various magnifications (10 and 100 kx). The FESEM images of MgO powder (Figures 3(a) and 3(b)) distinctly display the presence of crystalline NPs characterized by a polyhedral structure, with some evidence of agglomeration while maintaining uniformity and density. The observed aggregation of these NPs may be attributed to the electrostatic attraction among the MgONPs. It is worth noting that the polyhedral particles exhibit well-defined facets, with a majority of them being hexagonal crystals.

Figure 3(c) reveals that the diameters of the polyhedra, calculated from the images, were approximately 59 nm, which is consistent with the findings from the XRD analysis. In addition, Figure 3(d) demonstrates the qualitative and quantitative analyses of the elemental structure and purity of the MgONPs using EDX spectroscopy (EDX or EDS). The corresponding EDS spectra display strong peaks, indicating the presence of magnesium (Mg) and oxygen (O) elements in the sample without any impurities. The aforementioned analysis verifies the purity of the MgONPs. To gain further insights into the presence and distribution of elements in the sample, elemental mapping images of MgONPs were obtained. Figure 3(e) distinctly illustrates the presence of metallic magnesium atoms (blue color) and nonmetallic oxygen atoms (yellow color). Based on these images, it can be concluded that the presence of magnesium and oxygen atoms corresponds to the presence of MgONPs.

### 3.2. Cytotoxicity Assay

Based on the results of the cytotoxicity test, no toxic effects were observed on Vero cells at all concentrations of MgONPs (up to a concentration of 1000 μg/mL).

### 3.3. Antiviral Assays

#### 3.3.1. Virucidal Assay

**Figure 4:**
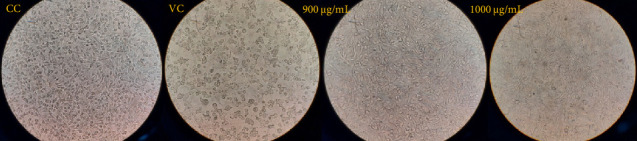
Microscopic images showing the inhibition of herpes simplex virus Type 1 (HSV-1)–induced cytopathic effects (CPEs) in Vero cells following a 3 h incubation of HSV-1 with MgONPs at concentrations of 900 and 1000 μg/mL in the virucidal assay. Images include a cell control (CC, uninfected Vero cells) and a virus control (VC, HSV-1-infected cells without MgONPs) for comparison.

Incubation of HSV-1 with MgONPs at both concentrations, 900 and 1000 μg/mL, for three hours was associated with remarkable reductions in the formation of CPEs (Figure 4).

**Figure 5:**
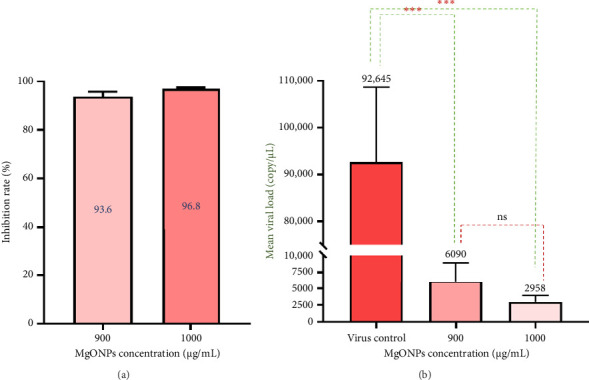
(a) Bar graph illustrating the percentage of HSV-1 viral inhibition in the virucidal assay, calculated by comparing viral loads in MgONP-treated groups (900 and 1000 μg/mL) to the virus control (VC) after a 3 h incubation, showing inhibition rates of 93.6% and 96.8%, respectively. (b) Bar graph depicting the corresponding HSV-1 viral loads (genomic DNA copies) in the virucidal assay, measured by quantitative real-time PCR, with significant reductions in treated groups compared to the virus control. ^∗∗∗^indicate *p* < 0.001, and “ns” means not significant.

The results of qPCR assay also showed that three-hour incubation of HSV-1 with MgONPs at concentrations of 900 and 1000 μg/mL resulted in a decrease in the number of copies of HSV-1 genomic DNA with an inhibition rate of 93.6% (*p* value < 0.001) and 96.8% (*p* value < 0.001), respectively, compared to the virus control. Accordingly, MgONPs showed their antiviral activity in a dose-dependent manner (Figure 5).

#### 3.3.2. Posttreatment Assay

**Figure 6:**
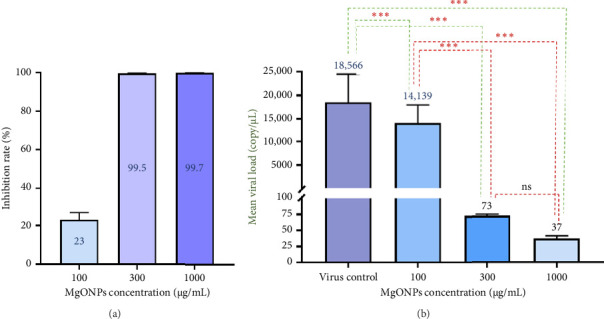
(a) Bar graph showing the percentage of HSV-1 viral inhibition in the posttreatment assay, calculated by comparing viral loads in MgONP-treated groups (100, 300, and 1000 μg/mL) to the virus control (VC) after 48 h of incubation, with inhibition rates of 23%, 99.5%, and 99.7%, respectively. (b) Bar graph depicting the corresponding HSV-1 viral loads (genomic DNA copies) in the posttreatment assay, measured by quantitative real-time PCR, demonstrating dose-dependent reductions in treated groups compared to the virus control. ^∗∗∗^indicate *p* < 0.001, and “ns” means not significant.

Following virus entry into the cells, the viral load was reduced in comparison to the virus control after 48 h of incubation with concentrations of 100, 300, and 1000 μg/mL of MgONPs. Based on the calculated viral loads and comparing them with the virus control, the percentage of viral inhibition by MgONPs at concentrations of 100, 300, and 1000 μg/mL was 23%, 99.5%, and 99.7%, respectively (*p* value < 0.001) (Table 1). Similar to the virucidal assay, MgONPs showed their antiviral activity in a dose-dependent manner in the posttreatment assay (Figure 6).

## 4. Discussion

To assess the antiviral effects of MgONPs against HSV-1 in this work, concentrations that maintained over 90% cell viability were utilized. As a result, all tested concentrations in the neutral red uptake assay were included in the antiviral tests since they were able to completely preserve cell viability. Therefore, the cytotoxicity test findings revealed that MgONPs exhibited a very high level of biocompatibility with cells and had no detrimental impacts on living cells. Thus, they can be considered a highly safe nanomaterial.

The direct effect of MgONPs on viral particles is evaluated in virucidal activity assessments, whilst the effect of MgONPs on various stages of viral replication is examined in posttreatment activity assessments. Our study found that the addition of MgONPs after cell infection considerably decreased the viral load of HSV-1, especially at the highest concentrations (300 and 1000 μg/mL), showing the largest antiviral effects. However, a lower concentration (100 μg/mL) of MgONPs was associated with a lower reduction in HSV-1 viral load, indicating that the antiviral action of MgONPs is dose-dependent.

Numerous studies have been conducted to investigate the antibacterial characteristics and efficacy of MgONPs [30–33]. However, only one study has so far precisely studied the antiviral activities of MgONPs. That study looked at the antiviral activity of MgONPs against FMDV [34]. The results of Rafiei et al. showed that MgONPs could decrease viral activity by more than 90% in the early phases of infection, including attachment and penetration. However, after the virus entered the cells, no antiviral effects were seen. At 3 and 6 hours after attachment, they added the NPs to infected cells to evaluate their influence on the viral replication cycle; however, no discernible change was seen at these phases of the FMDV infection cycle. These results imply that MgONPs prevent viruses from entering cells. Our research showed that treatment of HSV-1-infected cells with MgONPs, particularly at the higher doses, resulted in considerable reductions in the CPE and HSV-1 viral load compared to the virus control, which is in contradiction to the findings of the study by Rafiei et al. The timing of MgONP administration may be responsible for the difference in outcomes between the two studies. Unlike the study by Rafiei et al., where MgONPs were added to the cells at least 3 h after viral infection, our study added MgONPs to the cells 1 h after viral infection. It is important to remember that some viral replication mechanisms take place in the initial hours after infection. Therefore, in the study performed by Rafiei et al., the early stages of viral replication within the first 3 h may not have been influenced by MgONPs. This may explain why MgONPs did not show any effectiveness in their posttreatment analysis of viral infection.

Another explanation for the discrepancy between the results of the present study and those of Rafiei et al. could be the fact that Rafiei et al. evaluated the effects of MgONPs on a nonenveloped virus, whereas our investigation investigated the effects of these NPs on an enveloped virus. The absence of a lipid envelope makes nonenveloped viruses, such as FMDV, generally more resistant to environmental stressors [35, 36]. As opposed to this, the lipid bilayer membrane of enveloped viruses (such as HSV-1) is essential to their infectivity [37]. Enveloped viruses may be especially vulnerable to MgONPs because their lipid membrane is a target for oxidative stress.

The findings of our investigation indicate that the viral load has decreased when the virus was incubated with MgONPs for 3 h outside of the cellular environment. The results of this study demonstrate that MgONPs can directly affect virus particles. MgONPs and viral capsid proteins may directly interact to cause structural alterations that hinder viral attachment or entrance into host cells. Another portion of our study results has indicated that the addition of MgONPs after the entry of viruses into cells leads to inhibitory effects on the viral load. This result implies that MgONPs may employ other modes of action against HSV-1 in addition to direct impacts on the structure and viral particles.

MgONPs have been demonstrated to cause the production of intracellular reactive oxygen species (ROS) [38]. The uncontrolled ROS formation is thought to be caused by the release of free Mg2+ ions from the NP [39]. Previous research has demonstrated that the antibacterial activities of MgONPs are related to the generation of ROS. These ROS lead to lipid peroxidation, protein denaturation, bacterial cell death, intracellular leakage, and oxidative DNA damage [17, 32, 38]. Given the influence of MgONPs on bacterial cell membranes, it is reasonable to speculate about their effect on the structure of HSV-1. ROS and oxidative stress may cause damage to viral genetic material and proteins, making the virus noninfectious. MgONPs have the capacity to degrade the protein capsid structure or viral envelope of HSV-1, resulting in the disruption of its structure and a decrease in viral infectious titer. MgONPs may impair vital HSV-1 enzymes, hence impeding intracellular virus replication.

## 5. Conclusion

For the first time, the antiviral activity of MgONPs against HSV-1 was examined in this study. The data showed that MgONPs, especially at the highest concentrations, have no harmful impact on cell viability. In addition, there were notable inhibitory effects on HSV-1 from both direct exposure of the NPs to the virus and the addition of MgONPs following cell infection, especially at higher concentrations. These data indicate that MgONPs may be regarded as a promising and safe antiviral agent against several diseases caused by HSV-1. However, additional research on experimental models and laboratory animals is required to attain this goal.

## Figures and Tables

**Table 1:** Comparison of the antiviral activity between different concentrations of MgONPs against HSV-1 in the virucidal and posttreatment assay.

Setting	Compared groups	Average difference (%)	CI (%)	*p* value
Posttreatment	100 μg/mL vs. VC	23	17.9–28.0	< 0.001^∗^
	100 vs. 300 μg/mL	76.5	70.7–82.3	< 0.001^∗^
	100 vs. 1000 μg/mL	76.7	71.6–81.7	< 0.001^∗^
	300 μg/mL vs. VC	99.5	94.5–104.6	< 0.001^∗^
	300 vs. 1000 μg/mL	0.2	−5.2–4.8	0.99
	1000 μg/mL vs. VC	99.7	94.7–104.8	< 0.001^∗^

Virucidal	900 μg/mL vs. VC	93.6	90.8–96.4	< 0.001^∗^
	900 vs. 1000 μg/mL	−3.2	−6–(−0.3)	0.03^∗^
	1000 μg/mL vs. VC	96.8	94.0–99.6	< 0.001^∗^

Abbreviation: CI, confidence interval.

^∗^Differences with *p* values < 0.05 were considered significant.

## Data Availability

The data that support the findings of this study are available on request from the corresponding author. The data are not publicly available due to privacy or ethical restrictions.
